# Surrogates for myocardial power and power efficiency in patients with aortic valve disease

**DOI:** 10.1038/s41598-019-52909-9

**Published:** 2019-11-11

**Authors:** Chong-Bin Lee, Leonid Goubergrits, Joao Filipe Fernandes, Sarah Nordmeyer, Christoph Knosalla, Felix Berger, Volkmar Falk, Titus Kuehne, Marcus Kelm

**Affiliations:** 1 Charité – Universitätsmedizin Berlin, Institute for Computational and Imaging Science in Cardiovascular Medicine, Berlin, Germany; 2 German Heart Centre Berlin (Deutsches Herzzentrum Berlin), Department of Congenital Heart Disease, Berlin, Germany; 3 DZHK (German Centre for Cardiovascular Research), Partner Site Berlin, Berlin, Germany; 4 German Heart Centre Berlin (Deutsches Herzzentrum Berlin), Department of Cardiothoracic and Vascular Surgery, Berlin, Germany

**Keywords:** Cardiovascular biology, Valvular disease

## Abstract

We aimed to assess surrogate markers for left ventricular (LV) myocardial power and efficiency in patients with isolated aortic stenosis (AS) and combined stenosis/regurgitation (AS/AR). In AS (n = 59), AS/AR (n = 21) and controls (n = 14), surrogates for LV myocardial power and circulatory/external myocardial efficiency were obtained from cardiac MRI. Median surrogate LV myocardial power was increased in AS, 7.7 W/m^2^ (interquartile range 6.0–10.2; p = 0.010) and AS/AR, 10.8 W/m^2^ (8.9–13.4; p < 0.001) when compared to controls, 5.4 W/m^2^ (4.2–6.5), and was lower in AS than AS/AR (p < 0.001). Surrogate circulatory efficiency was decreased in AS, 8.6% (6.8–11.1; p < 0.001) and AS/AR, 5.4% (4.1–6.2; p < 0.001) when compared to controls, 11.8% (9.8–16.9). Surrogate external myocardial efficiency was higher in AS, 15.2% (11.9–18.6) than in AS/AR, 12.2% (10.1–14.2; p = 0.031) and was significantly lower compared to controls, 12.2% (10.7–18.1) in patients with reduced ejection fraction (EF), 9.8% (8.1–11.7; p = 0.025). In 16% of all cases, left ventricular mass/volume indices and EF were within normal ranges, wheras surrogate LV myocardial power was elevated and patients were symptomatic. Although influenced by pressure/volume load, the myocardium is additionally affected by remodelling processes. Surrogates for circulatory efficiency and LV myocardial power gradually reflect alterations in patients with AS and AS/AR, even when surrogate external myocardial efficiency, EF, mass and volume indices still remain compensated.

## Introduction

In aortic valve disease (AVD) pressure-volume overload conditions trigger cardiac remodeling causing concentric or eccentric hypertrophy^1–3^. The degree of hypertrophy is conventionally described by muscle mass and chamber size. However, large variability exists, how patients respond to pressure-volume overload and neither the onset of clinical symptoms nor the degree of hypertrophy (chamber size, wall thickness) are exclusively and directly related to the degree of external load^3,4^.

If left untreated, hypertrophy can lead to irreversible heart failure^1,2,5^ and arrhythmia with an increased risk for sudden cardiac death can evolve^6,7^. Methods that can help to better recognize the underlying mechanisms are therefore of high clinical relevance. There is growing evidence, that in valvular heart disease myocardial efficiency is reduced^8,9^. Hence, the myocardium will need more energy to pump a given amount of blood against the vasculature^8–10^. New pharmacological or interventional therapeutic concepts aim to enhance myocardial efficiency and/or reduce energy demand likewise^11^.

From a pathophysiological perspective, alterations in heart disease can occur at the mechanical and biochemical level^12–14^. Increased energy requirements are known to result in adaptive changes in myocardial mass, left ventricle (LV) chamber size and interstitial fibrosis in order to maintain the pump function of the heart. Furthermore, in the resulting mechanisms of hypertrophy, the ventricle requires more energy for contraction and becomes less energetically efficient^15,16^. These changes are associated to stiffening of the heart and to decreased efficiency of the LV^13,14^. Underlying biochemical processes include an increase in cellular energy demand in the hypertrophied heart^12,17^ and a metabolic switch from mitochondrial fatty acid oxidation to anaerobic glycolysis^15,16^.

The assessment of cardiac energy expenditure is challenging. Available methods are either invasive or associated to ionizing radiation and thus are limited in their clinical use^18,19^. In addition, most existing methods also measure cardiac work/energetics at different levels that are not directly comparable to each other. For example, conductance catheter techniques focus on the assessment of “mechanical” circulatory energy components whereas combined Magnetic Resonance Imaging (MRI)^20^ and Positron Emission Tomography (PET) methods have been used to describe energy efficiency between metabolic (biochemical) measures in relation to circulatory output (mechanical)^8,9^.

In this study, the aim was to assess surrogate markers for LV myocardial power and the resulting efficiency, using a non-invasive MRI technique without direct measurements of biochemical or cellular mechanisms of energy consumption. The objective was to apply this method in a cohort of patients with isolated aortic stenosis (AS) and combined aortic stenosis/regurgitation (AS/AR) in order to assess potential differences of these surrogate markers between groups.

## Materials and Methods

### Study population and design

The study was conducted in 80 patients with aortic valve disease (AVD) and 14 heathy volunteers. AVD patients were assigned to two groups: Patients with isolated aortic stenosis (n = 59), patients with combined aortic stenosis and regurgitation (n = 21). Furthermore, patients were compared to a group of volunteers (n = 14). The aortic stenosis (AS) group included patients with moderate or severe stenosis (mean gradient ≥20 mmHg)^21^ in the absence of moderate or severe aortic regurgitation (regurgitation fraction, RF < 30%). The AS/AR group included patients with moderate and severe AR (RF ≥ 30%)^22,23^ in the presence of AS. The control group included participants without any type of aortic valve disease. Ejection fraction (EF) sub-analyses were performed in patients with reduced (EF < 50%, n = 12) and normal EF (EF > 50%, n = 68). In patients where NT-pro-BNP laboratory data was available, information were included in the dataset (n = 32).

The pressure gradient across the aortic valve was assessed using Doppler echocardiography. Cuff-based blood pressure measurements were obtained from the patient’s right arm before Magnetic Resonance Imaging (MRI). Clinical symptoms of heart failure were assessed using the New York Heart Association (NYHA) classification. In controls, an identical protocol was applied, and the absence of AS was confirmed using four-dimensional velocity encoded MRI (4D-VEC MRI). Age and gender specific reference values from healthy volunteers^24^ were used to assess the presence or absence of abnormalities in parameters typically associated with left ventricular (LV) remodeling: LV muscle mass per body surface area (hypertrophy), LV end-diastolic and end-systolic volumes per body surface area (dilatation), LV EF. Measurements were considered within normal ranges if they were within two standard deviations of reference values^24^. An ejection fraction <50% was defined as functional impairment.

### Cardiovascular magnetic resonance and post processing

MRI examinations were performed using a whole body 1.5 Tesla MR system (Achieva R 3.2.2.0, Philips Medical Systems, Best, The Netherlands) using a five-element cardiac phased array coil. All MRI examinations lasted 45 to 60 minutes and were performed successfully.

Epicardial and endocardial segmentations as well as LV volumetry and anatomical measurements were performed based on previously described gapless balanced Turbo Field Echo (bTFE) cine two-dimensional short axis sequences^25^. All images were analyzed using View Forum (Philips Medical Systems Nederland B.V; View Forum R6.3V1L7 SP1). The entire ventricle and the myocardium without the papillary muscles were segmented during diastole and systole.

According to clinical standards aortic regurgitation was quantified in the ascending aorta distally to the valve using (a) two-dimensional phase contrast MRI: repetition time (TR) 3.9msec, echo time (TE) 2.4msec, flip angle (FA) 15°; 30time steps, voxel size 1.1 × 1.1 mm. Furthermore, (b) Four-dimensional velocity-encoded MRI (4D-VEC MRI) was used to quantify blood flow across the aortic valve, the mitral valve and the ascending aorta in order to assess auxobaric contraction time t_ABC_, isovolumetric contraction time t_IVC_ and the aortic pressure gradient, respectively: acquired voxel 2.5 × 2.5 × 2.5 mm, reconstructed voxel 1.7 × 1.7 × 2.5 mm, TR 3.5msec, TE 2.2msec, FA 5°, 25 reconstructed cardiac phases, retrospective gating, one signal average. 4D data were analyzed using GT Flow (Version 2.0.10, Gyrotools, Zurich, Switzerland). Total systolic contraction time t_CS_ is the sum of t_ABC_ and t_IVC_ measurements obtained directly from 4D flow.

### Quality assurance

High intra- and interobserver reproducibility of MRI-based volumetric LV and 2D flow measurements has been demonstrated^26–29^. Previous studies have also shown the accuracy and reproducibility of 4D-VEC MRI-based flow measurements and demonstrated good agreement to 2D flow data^30,31^.

As an alternative to 2D flow MRI, contraction time assessment included the quantification of the time to maximum aortic flow from 4D flow MRI sequences with 25 phases in order to allow for an optimal angulation (orthogonal). To exclude any methodological bias and to ensure sufficiently resolved data we performed a quality experiment in 15 aortic flow curves by comparing time measurements to higher temporal resolution 2D flow measurements with a temporal resolution of 75 phases. High temporal resolution flow curves were down-sampled to the resolution of the 4D flow MRI (25 phases) and the error for the peak flow time was evaluated. The median absolute error was 9.12 ms (interquartile range 9.1ms-18.2 ms) and thus was within acceptable range in our patients regarding an averaged t_CS_ of 174.5 ms (error <6%), making 4D flow measurements with 25 phases a feasible alternative to higher temporal resolution 2D flow if e.g. further post-processing is needed.

### Surrogate for LV myocardial power

**Figure 1:**
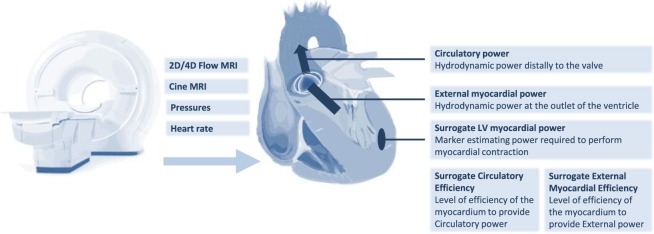
Summary illustrating the concept of surrogate LV myocardial power and power efficiency.

The surrogate for left ventricular myocardial power estimates the power of the LV myocardium required to perform contraction generating LV peak systolic pressure during a contraction time^32^. Since the applied method is an estimation, we defined it as a surrogate for myocardial power. The surrogate marker is a dimensional parameter [Watt] calculated as a product of the three parameters - myocardial wall stress σ_*wall*_, myocardial volume *V*_*wall*_ and the contraction time. Surrogate left ventricular myocardial power, sLVMP, was calculated in our study using the equation:

$$sLVMP=\frac{{V}_{wall}\,\ast \,{{\rm{\sigma }}}_{wall}}{{t}_{CS}}$$

in which V_wall_ is myocardial wall volume, σ_wall_ is peak systolic wall stress, and t_CS_ is the systolic contraction time (period between start of the contraction to peak systole) of the left ventricle. Peak systolic wall stress σ_wall_ was defined as:

$${{\rm{\sigma }}}_{wall}={P}_{sys}\,\ast \,\frac{{R}_{BP}}{2\,\ast \,{S}_{wall}}$$

where S_wall_ is the mean systolic myocardial wall thickness, P_SYS_ is the peak systolic pressure in the left ventricle and R_BP_ is the mean radius of the blood pool in peak systole. In order to correct for potential regional differences, mean myocardial wall thickness and mean radius of the blood pool estimations during peak systole were based on myocardial segmentations considering the LV as a cylindrical geometry. We then used a spherical Laplace-based approach as a very simplified model to estimate wall stress. Compared to finite element models, the combination with the spherical approach was demonstrated to provide a good approximation of the global mean stress in the circumferential‐longitudinal plane of the LV^33^. P_sys_ is the sum of the systolic pressure in the right arm and the peak systolic pressure gradient across the aortic valve. Surrogate LV myocardial power was indexed to body surface area (BSA) allowing inter-individual comparison. The concept of the surrogate LV myocardial power and power efficiency is summarized in Fig. 1.

### Circulatory power and surrogate power efficiency

Circulatory power defines the hydrodynamic power distally to the valve and represents the power needed to maintain effective blood flow against systemic vascular resistance (Fig. 1). Circulatory power, CP, was calculated as followed:

$${\rm{CP}}={\rm{MAP}}\,\ast \,{\rm{Q}}$$

where MAP is the mean arterial pressure and Q is the effective Cardiac Output (CO_eff_): CO_eff_ = (forward flow volume – backward flow volume) * HR (heart rate). The ratio between circulatory power and the surrogate LV myocardial power (CP/sLVMP*100 [%]) is defined as the surrogate circulatory efficiency (sCircE) of the heart.

### External myocardial power and surrogate power efficiency

External myocardial power defines the hydrodynamic power at the outlet of the LV (Fig. 1). It represents the power needed to maintain effective blood flow against all resistances distally to that site including the pressure gradient across a stenotic valve and the additional volume load due to regurgitation. External myocardial power, EMP, was computed by following equation:

$${\rm{EMP}}=({\rm{MAP}}+{\mathrm{mean}\Delta p}_{{\rm{valve}}})\,\ast \,{{\rm{CO}}}_{{\rm{total}}}$$

where meanΔp_valve_ is the pressure gradient across the aortic valve in peak systole and CO_total_ is the total (forward flow) stroke volume (SV_total_) multiplied by HR. The ratio between EMP and the surrogate LV myocardial power (EMP/sLVMP*100 [%]) was defined as surrogate external myocardial efficiency (sEME). sEME is the surrogate parameter for the level of efficiency of the myocardium to provide effective cardiac output against the diseased valve and systemic pressure.

### Statistics

Data are presented as median and interquartile range (Q1; Q3) unless stated otherwise. Shapiro-Wilk, Shapiro-Francia and Kolmogorov-Smirnov tests were used to test data for normality. Analysis of covariance (ANCOVA) in conjunction with Bonferroni correction was used as appropriate to adjust inter-group tests (myocardial power and efficiency) for age, gender as well as the presence of bicuspid valves. Dunn’s test was used as an appropriate nonparametric pairwise multiple comparison procedure of baseline data following a Kruskal-Wallis test of stochastic dominance among groups – it also included Bonferroni correction of p-values. Pearson’s chi-squared test was used in conjunction with Fisher´s exact test to test for differences in categorical data. Robust regression was used to assess multifactorial effects (and their 95% confidence interval, CI). The significance level was set at 0.05. Data were analyzed using Stata (Version 15.1, StataCorp, Texas, USA).

### Ethics approval and consent to participate

The study was carried out according to the principles of the Declaration of Helsinki and approved by the local ethics committee (Ethics committee - Charité Universitätsmedizin Berlin). Written informed consent was obtained from the participants and/or their guardians. Trial Registration: clinicaltrials.gov NCT03172338, June 1, 2017.

## Results

The analysis was performed in all included participants (N = 94). Table 1 shows a summary of general demographic and clinical parameters in both disease groups and controls. Significant disease-specific differences in geometrical and functional parameters were observed between groups (Table 2): Parameters of left ventricular hypertrophy including BSA-indexed myocardial volume, myocardial mass, left ventricular end systolic diameter (LVESD), myocardial wall thickness and mass-volume index were increased compared to controls. In AS/AR, even higher myocardial volume, myocardial mass, LVESD, myocardial thickness and mass-volume index than in AS were observed. In contrast, in the AS group, there were no differences for end diastolic volume index (EDVI) and end systolic volume index (ESVI) compared to controls. Furthermore, total cardiac output (CO_total_) was the highest in patients with AS/AR while there were no significant differences for efficient cardiac output (CO_eff_) between the groups. No significant differences for heart rate were observed between the groups.

**Table 1:** General demographic and clinical data; median and lower and upper quartiles (Q1; Q3) and n(%).

Subjects	AS (n = 59)	AS/AR (n = 21)	Control (n = 14)	p value AS vs. AS/AR	p value AS vs. control	p value AS/AR vs. control	p value overall
Age (years)	65 (53; 72)	41 (18; 61)	27 (25; 47)	<0.001	0.001	1.0	<0.001
Male gender (n)	33 (56%)	16 (76%)	8 (57%)				0.253
body mass index (kg/m^2^)	26.4 (22.9; 29.1)	23.2 (21; 28)	21.4 (20; 22.5)	0.101	<0.001	0.111	<0.001
BSA (m^2^)	1.86 (1.69; 2.11)	1.89 (1.63; 2.0)	1.83 (1.67; 1.91)	0.732	0.382	0.957	0.473
Bicuspid (n)	15 (25%)	14 (67%)	1 (7%)				<0.001
Dyslipidaemia* (n)	18 (31%)	4 (19%)	0 (0%)				0.046
Diabetes mellitus (n)	7 (12%)	0 (0%)	0 (0%)				0.106
CCS III-VI (n)	5 (8%)	1 (5%)	0 (0%)				0.477
NYHA III-IV (n)	18 (31%)	4 (19%)	0 (0%)				0.046
Arterial hypertension (n)	44 (75%)	8 (38%)	2 (14%)				<0.001
Systolic blood pressure [mmHg]	135 (127; 151)	119 (111; 141)	118 (108; 120)	0.014	0.001	0.479	<0.001
Diastolic blood pressure [mmHg]	77 (68; 83)	68 (55; 71)	68 (64; 73)	<0.001	0.025	0.738	<0.001
Mean arterial pressure [mmHg]	97 (88; 104)	84 (76; 91)	85 (79; 88)	<0.001	0.002	1.0	<0.001
Mean aortic pressure gradient [mmHg]	48 (36; 61)	24 (18; 50)	2 (2; 3)	0.009	<0.001	<0.001	<0.001
RF [%]	9 (4; 17)	40 (36; 54)	1 (1; 3)	<0.001	0.002	<0.001	<0.001

BSA, body surface area; RF, regurgitation fraction; CCS, Canadian Cardiovascular Society; NYHA, New York Heart Association; *presence of hyperlipoproteinaemia, hypercholesterinaemia and/or hypertriglyceridaemia.

**Table 2:** Geometric and functional parameters in AS, AS/AR and controls.

Parameter	AS (n = 59)	AS/AR (n = 21)	Control (n = 14)	p value AS; AS/AR	p value AS; control	p value AS/AR; control	p value overall
Myocardial volume/BSA end systolic [ml/m^2^]	65.1 (52.7; 79.6)	82.1 (73.5; 93.1)	36.7 (31.4; 47.6)	0.001	<0.001	<0.001	<0.001
Myocardial mass/BSA [g/m^2^]	68.3 (56.5; 83.5)	86.2 (77.2; 97.8)	38.6 (33.2; 48.3)	0.001	<0.001	<0.001	<0.001
LVESD end systolic [mm]	32.9 (30.0; 37.6)	42.0 (38.6; 45.0)	33.5 (31.4; 36.9)	<0.001	1.0	<0.001	<0.001
Myocardial wall thickness end systolic [mm]	10.7 (9.7; 12.4)	10.4 (9.6; 11.2)	7.0 (6.6; 7.6)	0.733	<0.001	<0.001	<0.001
EDVI [ml/m^2^]	86.3 (78.2; 100.1)	150.1 (116.9; 197.4)	91.4 (80.6; 108.7)	<0.001	0.806	<0.001	<0.001
ESVI [ml/m^2^]	36.9 (28.7; 44.4)	63.6 (51.5; 87.3)	36.7 (29.9; 48.2)	<0.001	1.0	<0.001	<0.001
Mass-volume-index [g/ml]	0.75 (0.62; 0.89)	0.59 (0.52; 0.71)	0.43 (0.39; 0.47)	0.006	<0.001	0.005	<0.001
HR [bpm]	68 (59; 76)	67 (63; 73)	65 (60; 73)	1.0	0.734	1.0	0.784
CO_total [l/min]	6.4 (5.4; 8.0)	9.7 (8.6; 10.8)	6.5 (6.1; 7.0)	<0.001	1.0	<0.001	<0.001
CO_eff [l/min]	5.7 (4.8; 6.9)	5.5 (4.4; 6.3)	6.4 (6.1; 6.8)	0.419	0.215	0.059	0.120
Contraction time [ms]	184 (157; 210)	165 (150; 192)	145 (129; 167)	0.336	0.006	0.176	0.014

LVESD, left ventricular end systolic diameter; EDVI, end diastolic volume index; ESVI, end systolic volume index; HR, heart rate; CO, cardiac output.

### Surrogate LV myocardial power

**Figure 2:**
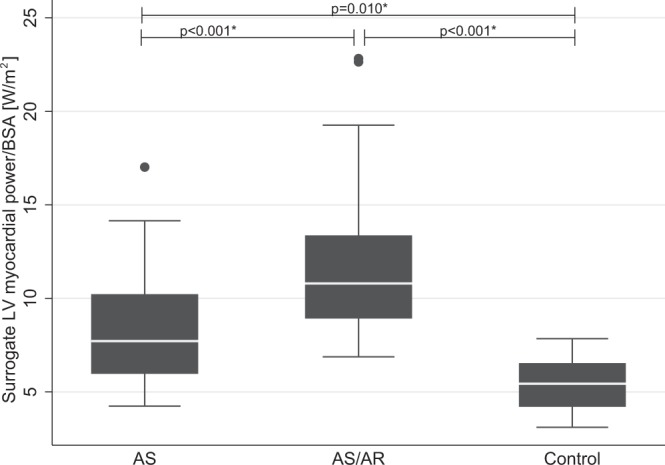
BSA-indexed surrogate LV myocardial Power in aortic stenosis, aortic stenosis/regurgitation and controls. *p-values adjusted for age and bicuspid aortic valve disease.

Figure 2 illustrates BSA-indexed surrogate LV myocardial power in AS, AS/AR and controls. BSA-indexed surrogate LV myocardial power was significantly higher in AS, 7.7 W/m2 (6.0–10.2; p = 0.010) and AS/AR, 10.8 W/m2 (8.9–13.4; p < 0.001) when compared to controls, 5.4 W/m2 (4.2–6.5), and AS was lower when compared to AS/AR (p < 0.001). In AS and AS/AR a multifactorial correlation (p < 0.001, R^2^ = 0.82) was found between surrogate LV myocardial power and: (1) indexed myocardial mass (Coef. 0.05 95% CI 0.02 to 0.08, p < 0.001) (2) indexed left ventricular end-diastolic volume (Coef. 0.06 95% CI 0.04 to 0.08, p < 0.001) (3) mean pressure gradient across the valve (Coef. 0.05 95% CI 0.03 to 0.07, p < 0.001), (4) age (Coef. 0.03 95% CI 0.01 to 0.05, p = 0.001) and (5) contraction time (Coef. −0.04 95% CI −0.05 to −0.03, p < 0.001). There was no significant correlation between surrogate LV myocardial power and sex (p = 0.087).

### Circulatory power and external myocardial power

Circulatory power and external myocardial power were assessed in all three groups. There were no significant differences for circulatory power in AS, 1.3 W (1.0–1.5; p = 1.0), and AS/AR, 1.0 W (0.8–1.2; p = 0.076) when compared to control group, 1.2 W (1.1–1.3). When comparing AS and AS/AR, circulatory power was higher in AS than in AS/AR (p = 0.003). External myocardial power was higher in AS, 2.1 W (1.7–2.6; p < 0.001) and in AS/AR, 2.4 W (2.1–2.9; p < 0.001) when compared controls, 1.3 W (1.2–1.3). There were no significant differences for external myocardial power between AS and AS/AR (p = 0.085).

### Surrogate power efficiency

**Figure 3:**
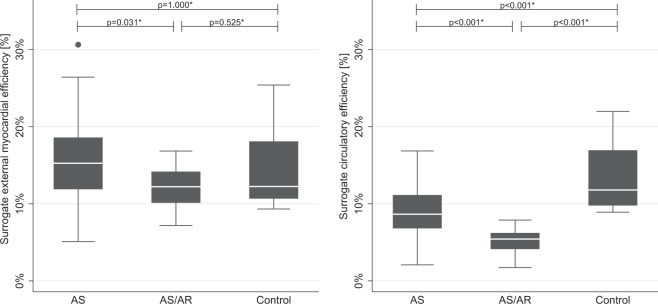
Power efficiency: External myocardial efficiency (EME) and Circulatory efficiency (CircE) in AS, AS/AR and controls. *p-values adjusted for age and bicuspid aortic valve disease.

Figure 3 illustrates surrogate circulatory and external myocardial efficiency in all three groups. As shown in Fig. 3, sCircE was significantly lower in AS, 8.6% (6.8–11.1; p < 0.001) and AS/AR, 5.4% (4.1–6.2; p < 0.001) when compared to controls, 11.8% (9.8–16.9), and AS was higher when compared to AS/AR (p < 0.001). sEME was significantly higher in AS, 15.2% (11.9–18.6) than in AS/AR, 12.2% (10.1–14.2; p = 0.031). There were no significant differences for sEME between AS and controls, 12.2% (10.7–18.1; p = 1.000) and AS/AR and controls (p = 0.525).

### Reduced ejection fraction

In AS and AS/AR, surrogate LV myocardial power was significantly higher in patients with reduced EF, 12.1 W/m2 (10.8–13.6) than in patients with normal EF, 7.9 W/m2 (6.4–10.5) (p < 0.001). sCircE was decreased in patients with reduced EF, 4.9% (4.1–5.9) compared to patients with normal EF, 8.0% (6.1–10.6) (p < 0.001). sCircE was lower in patients with normal EF (p < 0.001), and was lower in patients with reduced EF (p < 0.001), when compared to controls. sEME did not differ in patients with normal EF, 14.9% (12.9–17.9) when compared to controls (p = 0.218). sEME was significantly reduced in patients with reduced EF, 9.8% (8.1–11.7) when compared to patients with normal EF (p < 0.001) and compared to controls (p = 0.025).

### Correlation to established clinical standards

**Figure 4:**
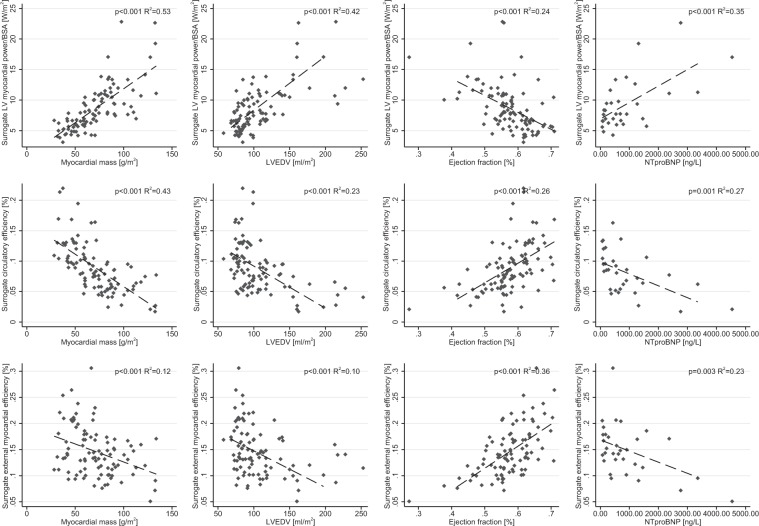
Correlations between surrogate LV myocardial power, Circulatory efficiency (CircE) and External myocardial efficiency (EME) to established clinical parameters: myocardial mass, LV end-diastolic volume (LVEDV), ejection fraction (EF) and N-terminal pro b-type natriuretic peptide (NT-proBNP).

In AS and AS/AR, there was a significant positive correlation between N-terminal pro b-type natriuretic peptide (NT-proBNP) and surrogate LV myocardial power (R^2^ = 0.37, p < 0.001). Furthermore, there were significant negative correlations between NT-proBNP and: (1) sCircE (R^2^ = 0.29, p = 0.001) and (2) sEME (R^2^ = 0.25, p = 0.003) in patients with AVD. Besides NT-proBNP, significant correlations between sLVMP, sCircE and sEME to established clinical standards for pressure-volume load assessment (LV mass/volume indices and EF) can be found (shown in Fig. 4). While correlations were significant, the coefficient of determination (R2) was below 54% for sLVMP and below 36% for sEME when compared to established parameters, with each of the single parameters only explaining some of the variability of the combined energetic measures.

Hypertrophy was present in 32/80 (40%), dilatation in 37/80 (46%) and an EF impairment in 12/80 (15%) patients. In 13/80 (16%) of all cases, left ventricular volume indices, EF and mass were within normal ranges^24^, wheras the surrogate LV myocardial power was elevated (above 6.8 W/m2) and patients were symptomatic (clinical symptoms during ordinary activity/less than ordinary activity or resting conditions).

## Discussion

Information about LV myocardial energy demand and efficiency are valuable for the understanding of heart disease^8–10^, including aortic valve disease where the heart is exposed to chronic pressure/volume overload. The patient-specific response to such overload conditions can vary substantially. Whereas some patients present with signs of hypertrophy, myocardial thickening and ventricular dilation, others may show alterations in contraction time or only modest signs of remodeling^3,4^. In addition to pressure/volume overload, these adaptive processes can contribute to changes in energy efficiency of the heart.

The study follows previous research, which found increased LV myocardial oxygen demand (MVO_2_) in hypertensive patients with LV hypertrophy^34^. The MVO_2_ is determined mainly by three factors: heart rate, velocity of contraction and systolic wall tension together with the myocardial mass^35^. The major part of myocardial energy is used for the contraction of the myocardial muscle and approximately 50% of the total MVO_2_ is required during the isovolumetric phase of contraction^35^. Experimental studies found that MVO_2_ correlates linearly with wall tension as well as the velocity of contraction, which inversely correlates to the generated tension^36,37^. This tension-velocity relationship is also affected by heart disease^38^. In order to estimate MVO_2_ in patients, recent studies proposed the use of a LV mass-wall stress-heart rate product and already demonstrated its association to mortality and heart failure in aortic valve stenosis^34,39^. The proposed surrogate LV myocardial power in our study can be of potential advantage, as (i) the use of myocardial volumes in our approach results in the power dimension [Watt] of the marker, that allows its direct use for the normalization of external and circulatory power and thus (ii) the calculation of the resulting surrogate efficiency. Furthermore, the integration of the contraction time instead of the heart rate (iii) corresponds with the major part of MVO_2_ demand^35^ and (iv) correlates better to the actual velocity of contraction.

In our study we used a non-invasive approach to quantify surrogate LV myocardial power and respective efficiency in a cohort of patients with isolated AS, combined AS/AR and controls. Surrogate LV myocardial power was increased in patients with aortic valve disease compared to controls, as what would be expected under conditions of increased afterload. In addition, sEME was at control levels in AS and AS/AR as long as the EF was preserved. However, sCircE was below control levels, even in patients with normal EF. The non-invasive character of this method made it possible to quantify these parameters in a routine clinical environment.

Several studies have shown that eccentric and concentric hypertrophy as well as an impairment of ventricular pump function can put patients with AS and AS/AR at risk for developing exercise intolerance, arrhythmia, sudden death and heart failure^1,2,5,6^. The degree of hypertrophy and pump dysfunction are typically quantified by ventricular volumes, muscle mass and EF. In addition to the presence of clinical symptoms, these parameters are used for medical decision making^24^. In our study, 16% of patients had ventricular volume indices, muscle mass and EF at normal levels, but at the same time exhibited clinical symptoms. All of these patients had markedly increased surrogate LV myocardial power. However, our study was not designed as an outcome study and further research is necessary to assess if such measurements can help to identify cardiac dysfunction at an early stage and whether they translate into disease progression and clinical outcome.

Cardiac energy demand and efficiency can be divided into biochemical and biomechanical components. PET and MR-spectroscopy^20^ are used to indirectly assess cellular metabolic (biochemical) parameters and have shown interesting – though not yet fully uniform – results in valvular heart disease. In an MRI-PET study, Güclü *et al*. have combined biochemical measures in a ratio with the individual’s mechanical external (circulatory) power in a small cohort of N = 10 patient. They demonstrate the resulting efficiency of the myocardium to be reduced in AS and a predictor of exercise capacity after aortic valve replacement^8^. In another combined MRI-PET study, Hansson *et al*. found reduced efficiency in patients with combined heart failure and low-flow low-gradient AS defined by indirect measurements of myocardial oxygen consumption in N = 59 patients^9^. In addition, the efficiency of compensated AS was reported to be at control levels. In our non-invasive approach, we also demonstrate (1) sEME to maintain normal in patients with compensated disease and (2) a similar decrease in sEME in patients with reduced EF. In our combined disease group (AS/AR), sEME was lower and surrogate LV myocardial power was substantially higher. These findings are in line with previous concepts of an accentuated degree of ventricular hypertrophy under combined pressure-volume load conditions^40^.

### The pathophysiology of elevated energy expenditure

Biomechanical stress due to pressure/volume overload triggers remodeling processes that lead to eccentric or concentric hypertrophy^1–3^. By the law of Laplace, an enlarged heart also attempts to maintain wall stress, amongst others, by changing its shape, increasing myocardial wall thickness or fibrous tissue content. By the same law, all adaptive changes necessarily go along with an increased energy demand. In line we noted in our study an increase in ventricular volume and mass (hypertrophy) to be an important determinant of the surrogate LV myocardial power. Further determinants were the pressure gradient and the regurgitation fraction across the aortic valve.

In patients with chronic heart failure exercise capacity can be reduced even before the onset of EF impairment^41^. Comparably, In AS/AI even when the EF is normal, sCircE is reduced as soon as ventricular mass, volume and contraction start to alter. During this gradual process, it would be of high interest to determine the tilting point from an efficient (adaptive) to an inefficient (maladaptive) myocardium. However, this question cannot be answered without performance of longitudinal studies.

Several biochemical cellular processes require energy for cell excitation, contraction, active relaxation and protein turnover^42^. Therefore, in heart disease, including AS/AI, it would be desirable to understand, to which extent and at which pace increases in energy demand cause energy starvation at the cellular level and in turn impair excitation-contraction coupling. Even if the proposed concepts of myocardial power and efficiency are still at an early stage, together with PET, spectroscopy and more detailed finite element approaches^33^ and further experimental research they open the possibilities for future studies, that combine mechanical concepts at the organ level with molecular studies at the cellular level.

### Methodological considerations

Earlier concepts for measuring parameters of myocardial energy demand and efficiency mainly required an indirect assessment of metabolic measures and/or involved the use of invasive methods. Conductance catheter derived pressure-volume-loops depict the external energy generated during one cardiac cycle and allow determining the mechanical efficiency of ventricular-arterial coupling^18,43,44^. More recent approaches applied combined MRI-PET methods. PET is used to determine the myocardial oxygen consumption that is considered being an estimate of myocardial energy utilization^8,9^. MRI-spectroscopy can assess metabolic processes, yet remains limited in its application as it typically requires higher magnetic field strengths. In the current study, we used a fully non-invasive MRI method without additional PET or spectroscopy with the advantage that it can be easily applied in clinical studies or even in the clinical routine. The method used in our study relies on an accurate and reproducible segmentation of the LV. Although echocardiography is able to assess flow, and thus can help estimating external power, it will be more dependent on image quality with larger heterogeneity between patients and observers, making the calculation of LV metrics and thus surrogate LV myocardial power more prone to errors. Nevertheless, recent and future technical improvements including the availability of 3D/4D echo may allow a translation to echocardiographic methods.

So far, we demonstrated a simplified approach for computing cardiac energetics. In this study, surrogate LV myocardial power was considered the potential energy of the LV myocardium generated by myocardial contraction and was based on geometrical parameters of the LV. Measures are, to some extent, comparable to those published in other studies. In a combined MRI-PET approach, Hansson *et al*. have shown AS patients are able to maintain normal energy efficiency until EF is reduced^9^, which corresponds to our findings regarding EME. In addition, the present MRI-based method permits assessing sCircE, that is able to detect imbalances between the circulatory power demand and the state of the myocardium, as soon as surrogate LV myocardial power increases due to alterations in underlying geometrical or functional measures.

Gsell *et al*. have described a detailed methodology how mechanical power can be assessed using a finite element (FE) approach^33^. The approach can have several advantages over the method presented here for the calculation of wall stress, as the FE method can be of high accuracy and considers the entire heart cycle as well as spatially resolved myocardial wall deformations. If segmentation and simulation resources as well as the necessary expertise are available, the method of Gsell *et al*. showed a benefit over more simplified Laplace law based approaches in 5 in-silico cases. External mechanical efficiency, using physically correct internal mechanical power, will be 100% in idealised conditions of FE simulations by Gsell *et al*. In our work a surrogate internal myocardial power is estimated, without the same physical meaning, and as such this fundamental mechanical balance does not hold.

In contrast, the here presented approach estimates the power of the LV myocardium required to generate such mechanical power during systole (contraction) and its efficiency respectively. The deviating idea of our approach is that during isovolumetric contraction and systolic ejection the major part of myocardial oxygen consumption takes places^35^. The energy for this will always be higher than the actual mechanical energy of the contraction. The loss of energy or higher MVO_2_ demand is a function of the contraction time, as faster contraction will be less efficient for the ventricle and it also accounts for differences found in AS and AS/AR compared to controls. Thus, the surrogate LV myocardial power as presented here is higher than the energy calculated by the approach of Gsell *et al*. When compared to circulatory power, the resulting ratio is defined as the surrogate circulatory efficiency. Whereas the different mechanical approaches carry the potential for clinical application, their correlation to biochemical and catheterization based methods as well as their comparative accuracy needs to be further investigated in future clinical studies.

### Limitations

While external power is based on mean arterial pressure and cardiac output (both commonly applied for pulsatile and non-pulsatile flow conditions in clinical routine), the surrogate LV myocardial power calculations are focused on the systole as this cardiac phase accounts for the majority of the heart’s energy expenditure. The surrogate LV myocardial power does not cover any direct metabolic measurements of myocardial energy consumption or MVO_2_ since the exact correlation between MVO_2_ LV myocardial volume, wall stress and the contraction time is not yet known. In contrast, PET at an aerobic state already allows quantifying oxygen consumed from the coronaries at the costs of using ionizing radiation. Additionally, surrogate LV myocardial power as evaluated in this study differs by definition from FE approaches that require wall stresses and myocardial wall strain rate^33^, as the approach by Gsell *et al*. represents the hydraulic pump power of the left ventricle, whereas surrogate LV myocardial power estimates the power of the LV myocardium required to perform contraction generating LV peak systolic pressure during a contraction time that also includes isovolumetric contraction (associated with zero resulting hydraulic pump power during this phase).

Both patient groups and controls were not matched and thus include differences, also reflecting disease specific characteristics, such as a high rate of patients with a bicuspid aortic valve. Group selection may have impacted relationships between surrogate LV myocardial power and power efficiency with the presented known clinical parameters. Comparison between groups was therefore statistically adjusted for age and bicuspid aortic valve disease. Nevertheless, genuine age/gender/disease specific reference cohorts should be acquired in future studies.

All aortic pressure gradients were assessed using Doppler echocardiography, according to current clinical recommendations. Consequently, pressure recovery was not considered and the aortic pressure gradient can be overestimated^45,46^ possibly overestimating the impact of the stenosis on surrogate LV myocardial power. It is suggested to improve the method in the future by using the continuity equation or model-based approaches^47^. The calculation of myocardial wall stress was simplified. Details of the geometrical shape of the LV as well as regional strain both impact myocardial wall stress and power and should be considered for more exhaustive and fine-tuned models. While higher temporal resolutions may be of help to improve contraction time measurements, their main benefit would be to increase the accuracy of the isovolumetric component, even this only accounts for 5–10% of total contraction time.

In addition, the present concept does not integrate processes at the tissue level such as fibrosis which is rather associated to diastole, as clinical routine myocardial mass measurements are also not corrected for extracellular volumes gained from T1 mapping. Future studies need to explore these mechanisms, especially as they may help to better understand the role of functional loss due to fibrotic remodeling and its impact on the remaining myocardium.

## Conclusions

While the predictive qualities and the impact on mid-/long-term outcome or progression to heart failure need to be further investigated, we were able to demonstrate that surrogate parameters of myocardial power and efficiency can be determined non-invasively using routine MRI data.

Although influenced by pressure/volume load, cardiac energetics is additionally affected by remodelling processes. As cardiac output needs to maintain stable, the surrogate circulatory efficiency starts to decrease from an early stage of the disease. Surrogate circulatory efficiency and LV myocardial power gradually reflect alterations in patients with AS and AS/AR, even when surrogate external myocardial efficiency, EF, mass and volume indices still remain compensated. This may add helpful information in future longitudinal studies and the evaluation of new treatment targets and strategies.

## Data Availability

The datasets used and analysed during the current study are available from the corresponding author on reasonable request.

## References

[1] Kupari M, Turto H, Lommi J (2005). Left ventricular hypertrophy in aortic valve stenosis: preventive or promotive of systolic dysfunction and heart failure?. Eur Heart J.

[2] Cioffi G (2011). Prognostic effect of inappropriately high left ventricular mass in asymptomatic severe aortic stenosis. Heart.

[3] Enache R (2010). CME: long-term outcome in asymptomatic patients with severe aortic regurgitation, normal left ventricular ejection fraction, and severe left ventricular dilatation. Echocardiography.

[4] Dweck MR (2012). Left ventricular remodeling and hypertrophy in patients with aortic stenosis: insights from cardiovascular magnetic resonance. J Cardiovasc Magn Reson.

[5] Dujardin KS (1999). Mortality and morbidity of aortic regurgitation in clinical practice. A long-term follow-up study. Circulation.

[6] Urena M (2015). Arrhythmia burden in elderly patients with severe aortic stenosis as determined by continuous electrocardiographic recording: toward a better understanding of arrhythmic events after transcatheter aortic valve replacement. Circulation.

[7] Wolfe RR (1993). Arrhythmias in patients with valvar aortic stenosis, valvar pulmonary stenosis, and ventricular septal defect. Results of 24-hour ECG monitoring. Circulation.

[8] Guclu A (2015). Myocardial efficiency is an important determinant of functional improvement after aortic valve replacement in aortic valve stenosis patients: a combined PET and CMR study. Eur Heart J Cardiovasc Imaging.

[9] Hansson, N. H. *et al*. Myocardial Oxygen Consumption and Efficiency in Aortic Valve Stenosis Patients With and Without Heart Failure. *J Am Heart Assoc* **6** (2017). 10.1161/JAHA.116.004810 PMC5523773 28167498

[10] Katz AM (1990). Cardiomyopathy of overload. A major determinant of prognosis in congestive heart failure. N Engl J Med.

[11] Eichhorn EJ, Bristow MR (1996). Medical therapy can improve the biological properties of the chronically failing heart. A new era in the treatment of heart failure. Circulation.

[12] Conway MA (1991). Detection of low phosphocreatine to ATP ratio in failing hypertrophied human myocardium by 31P magnetic resonance spectroscopy. Lancet.

[13] Alter P (2016). Wall stress determines systolic and diastolic function–Characteristics of heart failure. Int J Cardiol.

[14] Lieb W (2014). The natural history of left ventricular geometry in the community: clinical correlates and prognostic significance of change in LV geometric pattern. JACC Cardiovasc Imaging.

[15] Fillmore N, Mori J, Lopaschuk GD (2014). Mitochondrial fatty acid oxidation alterations in heart failure, ischaemic heart disease and diabetic cardiomyopathy. Br J Pharmacol.

[16] Tuomainen, T. & Tavi, P. The role of cardiac energy metabolism in cardiac hypertrophy and failure. *Exp Cell Res* (2017). 10.1016/j.yexcr.2017.03.052 28344054

[17] Jameel MN, Zhang J (2009). Myocardial energetics in left ventricular hypertrophy. Curr Cardiol Rev.

[18] Akins CW, Travis B, Yoganathan AP (2008). Energy loss for evaluating heart valve performance. J Thorac Cardiovasc Surg.

[19] Paul Knaapen, T. G. Myocardial efficiency in heart failure: non invasive imaging. *Heart and Metabolism* (2008).

[20] Hudsmith LE, Neubauer S (2009). Magnetic resonance spectroscopy in myocardial disease. JACC Cardiovasc Imaging.

[21] Baumgartner H (2009). Echocardiographic assessment of valve stenosis: EAE/ASE recommendations for clinical practice. Eur J Echocardiogr.

[22] Lancellotti P (2013). Recommendations for the echocardiographic assessment of native valvular regurgitation: an executive summary from the European Association of Cardiovascular Imaging. Eur Heart J Cardiovasc Imaging.

[23] Zoghbi WA (2003). Recommendations for evaluation of the severity of native valvular regurgitation with two-dimensional and Doppler echocardiography. J Am Soc Echocardiogr.

[24] Hudsmith LE, Petersen SE, Francis JM, Robson MD, Neubauer S (2005). Normal human left and right ventricular and left atrial dimensions using steady state free precession magnetic resonance imaging. J Cardiovasc Magn Reson.

[25] Fernandes JF (2017). Beyond Pressure Gradients: The Effects of Intervention on Heart Power in Aortic Coarctation. PLoS One.

[26] Olivotto I (2008). Assessment and significance of left ventricular mass by cardiovascular magnetic resonance in hypertrophic cardiomyopathy. J Am Coll Cardiol.

[27] Vogel-Claussen J (2006). Left ventricular papillary muscle mass: relationship to left ventricular mass and volumes by magnetic resonance imaging. J Comput Assist Tomogr.

[28] Grothues F (2002). Comparison of interstudy reproducibility of cardiovascular magnetic resonance with two-dimensional echocardiography in normal subjects and in patients with heart failure or left ventricular hypertrophy. Am J Cardiol.

[29] Noda C (2016). Reproducibility of functional aortic analysis using magnetic resonance imaging: the MESA. Eur Heart J Cardiovasc Imaging.

[30] van Ooij P (2016). Reproducibility and interobserver variability of systolic blood flow velocity and 3D wall shear stress derived from 4D flow MRI in the healthy aorta. J Magn Reson Imaging.

[31] Nordmeyer S (2010). Flow-sensitive four-dimensional cine magnetic resonance imaging for offline blood flow quantification in multiple vessels: a validation study. J Magn Reson Imaging.

[32] Preston, R. R. & Wilson, T. E. *Lippincott’s Illustrated Review: Physiology*. 203–213 (Lippincott Williams & Wilkins, 2012).

[33] Gsell MAF (2018). Assessment of wall stresses and mechanical heart power in the left ventricle: Finite element modeling versus Laplace analysis. Int J Numer Method Biomed Eng.

[34] Devereux RB (2015). Left Ventricular Wall Stress-Mass-Heart Rate Product and Cardiovascular Events in Treated Hypertensive Patients: LIFE Study. Hypertension.

[35] Alexios, S. A., Georgia Vogiatzi, A. G. & Tousoulis, D. *Coronary Artery Disease, From Biology to Clinical Practice*. 127–136 (2018).

[36] Hoffman JI, Buckberg GD (2014). The myocardial oxygen supply:demand index revisited. J Am Heart Assoc.

[37] Coleman HN (1967). Role of acetylstrophanthidin in augmenting myocardial oxygen consumption. Relation of increased O-2 consumption to changes in velocity of contraction. Circ Res.

[38] Ross J, Covell JW, Sonnenblick EH (1967). The mechanics of left ventricular contraction in acute experimental cardiac failure. J Clin Invest.

[39] Gerdts, E. *et al*. Higher left ventricular mass-wall stress-heart rate product and outcome in aortic valve stenosis. *Heart* (2019). 10.1136/heartjnl-2018-314462 PMC6855785 31154431

[40] Zilberszac R (2013). Outcome of combined stenotic and regurgitant aortic valve disease. J Am Coll Cardiol.

[41] Yap J (2015). Correlation of the New York Heart Association Classification and the 6-Minute Walk Distance: A Systematic Review. Clin Cardiol.

[42] Kolwicz SC, Purohit S, Tian R (2013). Cardiac metabolism and its interactions with contraction, growth, and survival of cardiomyocytes. Circ Res.

[43] Asanoi H, Sasayama S, Kameyama T (1989). Ventriculoarterial coupling in normal and failing heart in humans. Circ Res.

[44] Burkhoff D, Sagawa K (1986). Ventricular efficiency predicted by an analytical model. Am J Physiol.

[45] Amsel BJ (1997). Pressure recovery across the aortic valve. Acta Clin Belg.

[46] Bahlmann E (2010). Impact of pressure recovery on echocardiographic assessment of asymptomatic aortic stenosis: a SEAS substudy. JACC Cardiovasc Imaging.

[47] Donati F (2017). Beyond Bernoulli: Improving the Accuracy and Precision of Non-Invasive Estimation of Peak Pressure Drops. Circulation. Cardiovascular imaging.

